# Supplementary material for: Is there a hybridization barrier between Gentiana lutea color morphs?
Source: PeerJ. 2015 Oct 27;3:e1308. doi: 10.7717/peerj.1308 (PMC4627910; doi:10.7717/peerj.1308)
Supplement: Data S1 [file peerj-03-1308-s002.pdf]

	Levene's Test for Homogeneity of Variances (*Gentiana lutea*'s reproductive system)<br>Effect: "Treatment"<br>Degrees of freedom for all F's: 4, 125			
	MS<br>Effect	MS<br>Error	F	p
Nºovules	226,8064	380,3502	0,596309	0,665962

Effect	Univariate Tests of Significance for Nºovules (Sistema reprodutivo_2011_Torrestio_Plantas enteiras)<br>Sigma-restricted parameterization<br>Effective hypothesis decomposition				
	SS	Degr. of Freedom	MS	F	p
Intercept	*1591977*	*1*	*1591977*	*1489,054*	*0,000000*
Treatment	6369	4	1592	1,489	0,209382
Error	133640	125	1069		
